# A hospital-based independent domestic violence advisor service: demand and response during the Covid-19 pandemic

**DOI:** 10.1186/s12913-022-08183-z

**Published:** 2022-07-05

**Authors:** Rebecca Elvey, Thomas Mason, William Whittaker

**Affiliations:** The University of Manchester, Manchester, UK

**Keywords:** Domestic violence and abuse, COVID-19, Hospitals, Health services, Evaluation, Qualitative, Quantitative

## Abstract

**Background and aim:**

Recent UK policy has focussed on improving support for victims of domestic violence and abuse (DVA), in healthcare settings. DVA victims attending hospital are often at highest risk of harm, yet DVA support in hospitals has been inadequate. A targeted service supporting high risk DVA victims, was implemented at a hospital Trust in North West England. The service was provided by Independent Domestic Violence Advisors (IDVAs). This paper assesses the activity in the hospital-based IDVA service during the COVID-19 pandemicand addresses the research questions: What was the demand for the service? How did the service respond? What facilitated this response?

**Methods:**

A mixed-methods study was undertaken. Quantitative data on referrals to the service were examined using simple descriptive statistics and compared to other DVA services. Semi-structured interviews were undertaken with IDVAs and other hospital staff involved with the service and the data subjected to thematic analysis.

**Results:**

The quantitative analysis showed that referrals dropped at the start of lockdown, then increased and continued to rise; the qualitative findings reiterated this pattern. Referrals came from a range of departments across the Trust, with the majority from A&E. Pre-pandemic, the population supported by the service included higher proportions of males and people aged 40 and over than at other IDVA services; this continued during the pandemic. The qualitative findings indicated a flexible response during the pandemic, enabled by strong working relationships and by using workarounds.

**Conclusions:**

The hospital-based IDVAs provided an efficient, flexible serviceduring the COVID-19 pandemic. Referrals increased during the first lockdown and subsequent relaxing of restrictions. Locating the IDVAs within a team working across the organisation, and building good working relationships facilitated an effective disclosure and referral route, which endured through social restrictions. The IDVAs supported high-risk victims who may otherwise not have been identified in traditional community-based DVA settings during the pandemic. Hospital-based IDVA services can broaden access by supporting vulnerable, at risk populations whose needs may not be identified at other services.

## Background

Domestic violence and abuse (DVA) is a widespread problem, affecting an estimated one in three women worldwide during their lifetime [1]. In England and Wales, DVA is experienced by over two million people (5.7% of the population) every year and over 20% report having had at least one incident of DVA in their lifetime [2]. DVA includes any incident or pattern of incidents of controlling, coercive or threatening behaviour, violence or abuse between people aged 16 or over, who are (or were) family members or intimate partners. These behaviours include psychological, physical, sexual, financial and emotional abuse, ‘honour’-based violence and forced marriage [3]. DVA has serious, sometimes fatal consequences for victims and their families; it has detrimental impacts on physical and mental health and can have long term effects on social, emotional and behavioural functioning [4–6]. In terms of the wider social and economic impacts, the estimated costs of DVA are more than £66 billion per year (£34,015 per victim), including personal cost to victims, lost economic output, health and other public services such as policing and housing [5]. Thus, DVA is a common problem, with serious, wideranging impact.

In recent years, there has been an increased government policy focus in the UK, on tackling DVA. Strategic approaches have included both prevention and response. In terms of responding to victims at high risk of serious harm, the focus has been on targeted support provided by specialist workers [4]. A new role developled: Independent Domestic Violence Advisor (IDVA). IDVAs work with domestic violence victims, from the point of crisis, to assess risk, discuss suitable options and develop safety plans [7]. IDVAs now work in a range of organisations (Table 1). Whilst in community settings, specialist support such as IDVA services has become well established, in healthcare settings this is not the case; specialist DVA support is less developed and often lacking entirely. This is problematic, considering that victims of DVA tend to seek healthcare more frequently than those who have not experienced abuse [8–10]. When abuse is disclosed, if there is no appropriate service available to refer onto, the ability of healthcare staff to offer effective help is limited [11]. Furthermore, abuse often remains hidden, even when victims access healthcare, for example, victims often present seeking help for associated conditions, such as mental health problems, or physical injuries, caused by DVA, without disclosing it [3, 8, 12, 13]. The factors influencing non-disclosure are complex and include shame and embarrassment among victims, in addition to: lack of time, awareness, confidence, and skills/training amongst healthcare professionals [3, 8, 12, 13]. Thus, there is a need to improve rates of identification of DVA, in healthcare, as well as the response when it is disclosed.

**Table 1:** The IDVA role

The independent domestic violence advisor (IDVA) is a specialist practitioner role. In 2005, an accredited training course for IDVAs was established, which provided a formal qualification, framework for practice and service standards for practitioners. Most practitioners who obtain the IDVA qualification are those already working in ‘domestic violence practitioner’ roles, providing domestic violence services, they are not usually health care professionals. IDVAs are employed predominantly in community settings where many are based in voluntary organisations, such as specialist domestic abuse services, police forces, housing associations, Local Authorities and courts. It is estimated that there are over 1000 IDVAs in England and Wales at a cost of £25 million [5]. IDVAs support high risk DVA victims, often at the point of crisis, addressing immediate needs and providing intensive short to medium term support. They are pro active in implementing safety plans, which in the short term may include practical steps to protect victims and their children. The safety plans also include actions from the MARAC as well as sanctions and remedies available through the courts, housing services and resources available through other organisations.	

In line with insufficient provision across healthcare, DVA support in hospitals has been lacking. Inadequate hospital-based DVA support is particularly serious, as DVA victims attending hospital are often at the highest risk of harm [14]; women who have experienced more severe DVA are more likely to attend Accident & Emergency (A&E). Despite this, many hospitals have had no DVA policies or referral pathways, onsite hospital DVA services are rare and few referrals are made from hospitals to community DVA services [1, 8, 11]. Thus, there is clear potential to improve support for victims facing some of the most severe DVA.

One development aimed at improving specialist DVA support in hospitals has been the establishment of hospital-based IDVA services; IDVAs have worked from A&E, minor injuries, maternity and sexual health departments [12, 14–17]. Evaluations have found several benefits to delivering DVA support in this way – the addition of specialist DVA support and a referral route – within the hospital [12, 15]. Staff reported high satisfaction levels [16], increased confidence and skills in asking about and responding to DVA [12, 16]. Barriers to implementing and embedding services related to engaging staff across large hospitals and complex management/employment structures; some hospital-based IDVAs were seconded from and/or still line managed by third sector organisations, so competing priorities were a challenge [12, 15]. Evidence suggests these services can be effective, with increased DVA detection and referral rates following implementation [14, 16]. In a study of five hospitals, the majority of referrals came from A&E, followed by maternity and post/ante natal services and mental health/psychiatry [11]. Patients referred to hospital-based IDVAs had high rates of hospital attendance and admissions [14] and were more likely to be high risk cases [14] to be older, more affluent, more likely to be pregnant and identified earlier than at community IDVA services [11].

In early 2020, the COVID-19 pandemic spread across the UK. Restrictions intended to reduce transmission of the virus were introduced in England during March 2020: from the 23rd March there was a full national lockdown, which brought societal restrictions including a ‘stay at home’ order and social distancing requirements. Some restrictions were eased from May 2020; but in Greater Manchester in North West England, restrictions were increased again from 30th July (albeit to a lower level than during the full national lockdown). Concerns about DVA grew due to ‘stay at home’ orders, which meant households were largely confined to their own homes. With victims and perpetrators kept in close proximity, with reduced opportunities for victims to seek help [18], it was widely suggested that DVA would increase. Indeed, police recorded crime data showed a rise in DVA since the start of the pandemic [19] and demand for DVA charity services increased [20].

### Provision of a hospital-based IDVA service during the Covid-19 pandemic

In 2018, an IDVA service was implemented in a hospital Trust (the Trust) in North West England. See Table 2 for a description. Previously, an ‘IDVA pilot scheme’ had been run at the Trust, whereby an IDVA was seconded (from a voluntary organisation) and was based in A&E. The new service was designed to improve on the pilot; in particular, the IDVAs were directly employed by the Trust and were part of the safeguarding team, which works across the Trust. A team at the National Institute for Health and Care Research Applied Research Collaboration Greater Manchester (ARC-GM) was commissioned to evaluate the service. The evaluation was designed to investigate the processes and outcomes associated with the service. Whilst the COVID-19 focus was not established at the design stage, the evaluation coincided with the early stages of the pandemic and first national lockdown, a situation where DVA became an increasing concern. This coincidence provided an opportunity to investigate the impact of the pandemic on the service and the ways in which the service responded. To date, little evidence has been available on providing hospital-based IDVA services during the COVID-19 pandemic. Our aim, therefore, in this paper, was to explore and understand service activity and response during the pandemic. Our research questions were: What was the demand for the service? How did the service respond? What facilitated this response?

**Table 2:** Description of the hospital-based IDVA service

Item number	TIDieR Checklist element
**1**	**BRIEF NAME**
	Wrightington, Wigan and Leigh Teaching Hospitals (WWLTH) NHS Foundation Trust (the Trust) Independent Domestic and Sexual Violence Advocate (IDVA). A secondary care based intervention, aimed at providing enhanced support for victims of domestic and/or sexual violence and abuse at one hospital trust.
**2**	**WHY (rationale, theory or goals)**
	A need to improve rates of identification of domestic violence and abuse (DVA) was ascertained. Previously, DVA had a low profile in the Trust, and the response to victims had been insufficient, with no DVA trust policy or clear referral pathway. Staff often lacked awareness of the signs of DVA, confidence or skills to enquire or act when DVA was suspected or disclosed.
	For a previous IDVA pilot in the Trust, an IDVA was seconded from a voluntary organisation and worked in A&E. The employment arrangements for the current pilot aimed to improve on this, the direct employment of the IDVA meant that they were line managed entirely within the trust and their location within the safeguarding team was designed to improve their reach across the trust (deemed important in identifying victims) and to raise their profile (by engaging with staff across all departments).
	The IDVA service follows guidelines and pathways for responding to DVA, these recommend a dual approach, with: (i) specialist DVA services run by specialist staff with high levels of training in DVA; (ii) all staff having lower level DVA training, to increase awareness of the signs of DVA and skills to perform selective routine enquiries in a sensitive manner, so that when DVA is suspected or disclosed, referrals are made to specialist services [3, 11].
	The service is based on the safeguarding model ‘Triage and Make safe’ deemed suitable for the hospital environment; the IDVAs provide an immediate response and aim to work intensively with cases in a short timeframe then refer them elsewhere.
	The goals of the service are to:
	• Increase WWLFT staff awareness of the indicators of DVA
	• Increase identification of cases, through staff initiating sensitive routine enquiry of patients.
	• To ensure staff have the following skills and attributes: awareness of the indicators of DVA; sufficient professional curiosity to recognise suspected DVA; skills to initiate sensitive enquiry and respond to disclosures, including referral to adequate support.
**3 and 4**	**WHAT (process and materials)**
	Staff training
	The IDVAs provide structured training on DVA to Trust staff, this has become part of mandatory safeguarding training. The focus is on equipping staff with the skills outlined in section 2. The IDVAs also provide ongoing, ad hoc training and developmental support, e.g. updating staff on the outcomes of referrals, successes and areas for improvement such as when a case could have been identified earlier.
	Risk assessment, referral and case management
	*Process:*
	Cases are referred by Trust staff to the IDVA who performs a risk assessment. After the risk assessment, the patient is either (i) supported by the IDVA, (ii) referred to a local agency (ies), (iii) referred to the MARAC or (iv) declines support. Most patients return to their own homes, but some are discharged straight to refuges for their safety.
	The IDVAs work with the MARAC on a daily basis. They prepare referrals with the DASH and supporting information and present the case to the MARAC; if taken on they are usually supported by a community IDVA. The IDVA also liaise with voluntary organisations, local authorities, and the police. They provide various types of support: signposting, safety planning; support with applications (e.g. for housing) legal processes, arranging legal aid, completing paperwork if legal aid is unavailable, attending court with victims.
	*Materials:*
	The DASH form is completed for all risk assessments. The service is publicised via posters displayed in the hospital, with tear-off strips with contact details on.
**5**	**WHO PROVIDED**
	Two IDVAs, both experienced domestic violence practitioners (not healthcare professionals) completed formal IDVA training whilst working in the community. One is an experienced manager and a qualified Independent Sexual Violence Advisor (ISVA) was undertaking ISVA training.
**6**	**HOW**
	The staff training is provided by the IDVAs, to groups of staff at the Trust. Ongoing, ad hoc support and developmental feedback is provided to individuals.
	Staff often call the IDVA for immediate support when they have a patient with them, the IDVA comes and performs the risk assessment If the IDVA is unavailable, the staff member completes an initial risk assessment and sends it through the hospital safeguarding system, via email or an incident reporting system (for staff based outside the hospital e.g. walk in centre) or on paper (out of hours A&E). The IDVA picks up the referral as soon as possible and undertakes a full risk assessment.
**7**	**WHERE**
	• The service is based in an acute care organisation in North West England; a medium-sized NHS Foundation Trust with three hospital sites, a walk in centre and community services including health visiting.
	• Case identification happens on Trust premises and also in the community, for example, during a Health visitor appointment at the patient’s home.
	• Risk assessments and follow up appointments take place on the Trust premises, in a private room. The IDVAs also work outside the trust premises as necessary, for example attending court.
	• Previously, the MARAC took place at the local police station, since the COVID-19 restrictions it has been held via teleconference.
**8**	**WHEN AND HOW MUCH**
	The IDVAs provide an immediate response to DVA disclosures, going to the patient as soon as possible. One risk assessment is undertaken for each patient (unless a staff member performs an initial one in the absence of an IDVA then two are done). The service runs from 8 am to 6 pm Monday to Friday.
	Support is provided to the patient as required. One IDVA attends each MARAC, previously the MARAC was held once a week, currently it runs daily Monday to Friday.
	• In some cases the IDVA’s input ends soon after completion of the risk assessment – for patients who require brief input such as signposting to a relevant agency and for those who decline support.
	• Due to the range of presentations, the support provided varies in terms of length, intensity and nature and be long term. The IDVA retains cases which are members of staff at WWLFT.
	• The IDVAs retain all cases who are staff members at WWLFT and support them as long as necessary. They also support cases who do not meet the MARAC threshold but require ongoing support.
	• Some victims decline support initially and contact the IDVA after the consultation, when they feel ready to access support and/or when it is safe for them to do so; the IDVAs emphasise to victims that the service is available to them later on, not just immediately.
**9**	**TAILORING**
	The service is designed to provide tailored support.
**10**	**MODIFICATIONS**
	• The service initially operated with one full time IDVA post, in 2019 this increased to two posts.
	• In addition to domestic abuse, the service also received referrals for sexual assaults in a domestic abuse situation; this support has now been built into the service.

The evaluation report, including qualitative and quantitative findings from the full evaluation period is available at https://arc-gm.nihr.ac.uk/projects/project/Organising+Care/hidva-service-evaluation.

## Methods

We used a mixed methods approach, in which quantitative methods were used to explore service activity and qualitative methods were used to understand the processes through which the service responded. In order to ascertain demand for the service and to understand how this demand was met, a mixed methods approach was deemed appropriate [21]. We analysed quantitative data on service activity, to explore referral patterns before and during the pandemic and we generated qualitiative data, through interviews with staff at the Trust, to understand the processes involved with service provision. Our approach included using a ‘triangulation’ design; quantitative and qualitative study components had equal priority and were undertaken concurrently [22]. Two researchers worked separately to collect and analyse the data; TM undertaking the quantitative work and RE the qualitative, but the whole team met regularly to discuss the emerging analysis and through this process, we gained mutually enhanced understandings of the topic in question and additional insights that would not have been gleaned from the qualitative or quantitative findings alone [23]. In the methods and findings sections, we present the quantitative research first and the qualitative second and then bring them together in the discussion. A university ethics signatory confirmed that approval by an ethics committee was not required, on the basis that the study was a service evaluation. Also, the analyses did not include any personally identifiable data.

### Quantitative evaluation of referral activity

The Trust provided data on referrals to the hospital-based IDVA service from May 2018 to August 2020; this includes the first 2 years of the service, from 1st May 2018 to 31st March 2019 (year 1) and 1st April 2019 to 31st March 2020 (year 2) as well as from 1st April to 31st August 2020, that is, the first months of the COVID-19 pandemic in the UK, which for the purposes of this analysis, we define as the ‘COVID’ period. The full study period, including referrals to the service before the COVID period, was used to provide context and to allow assessment of whether activity changed during the emerging pandemic.

The data were analysed using simple descriptive statistics. Comparisons are made to referrals to IDVA services in England and Wales for the period April 2018 to March 2019 [24], this comparison sample consisted of 3672 referrals, spread across 22 IDVA services, which were mainly community-based, but included some hospital-based IDVA services. For the purposes of this paper we use the term ‘other IDVA services’. This comparison was made to identify whether the hospital-based IDVA service may be identifying a different type of victim than other IDVA services. The populations covered by the IDVA dataset may differ from those in Wrightington, Wigan and Leigh, therefore, comparisons were also made between populations.

### Qualitative process evaluation

Interviews were conducted between June and August 2020. Participants were sampled purposively, for their role in providing or referring to the hospital-based IDVA service, thus, we sought to interview the IDVAs and other (non-IDVA) staff within the safeguarding team as well as those in other teams at the Trust, who made referrals to the IDVA service. We identified potential interviewees through the service specification, key contacts for the evaluation and through ‘snowballing’ when we asked participants to suggest other interviewees. All interviews were conducted over the telephone, audio recorded with informed consent and fully transcribed, by a professional transcription company. A semi-structured interview schedule was used, with open-ended questions. Interviews focussed on the day to day running of the service, facilitators and challenges to its implementation. We conducted the interviews shortly after the first COVID-19 restrictions started, thus we also asked about the impact of the restrictions on the service. Data analysis followed a thematic approach, whereby categories were generated through repeated reading of the transcripts and discussion within the research team [25]. Themes were derived from the research questions and from the data themselves. The analysis presented here focussed on understanding experiences of providing the IDVA service during the COVID pandemic.

## Findings

### Quantitative findings

#### Referral rates, sources and outcomes

**Fig. 1:**
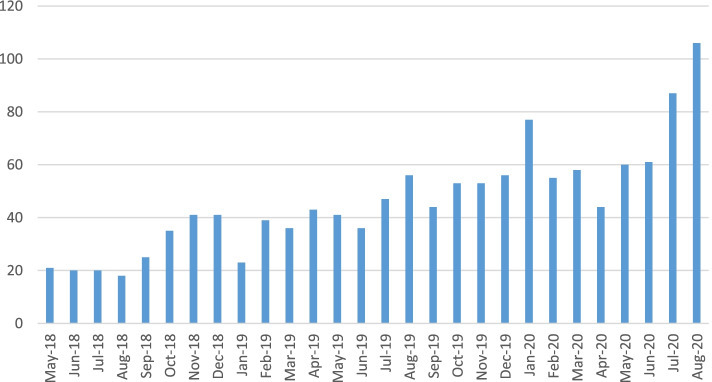
Referrals into the IDVA service 1st May 2018 to 31st August 2020

Referrals to the service (shown in Fig. 1) increased over time; during year 1 there were 319 referrals, during year 2 there were 619 and during the Covid period there were 358. Referrals dipped slightly in April 2020 but increased afterwards, rising to a new peak in August.

Table 3 presents the demographics of victims referred into the IDVA service over the first and second year of the service and the COVID-19 period. Rates are also provided from SafeLives Insights data on referrals to other iDVA services for comparison. 13% of referrals were male in the pre- COVID-19 period, in comparison, just 4% of referrals to other IDVA services were male. The proportion of referrals aged 40 or over, increased; from 33% in year 1, to 41% in year 2 and 43% during COVID-19; in other IDVA services, just 26% of referrals were aged 40 or over. During the COVID-19 period, there was little change in the demographics of victims referred overall. IDVA services appear to have a relatively greater share of referrals of black and minority ethnic (BAME) populations. Caution is advised with these figures as differences may reflect demographic differences between the Wrightington, Wigan and Leigh locality, and the IDVA services sampled in the SafeLives case notes assessment. For example, the Wrightington, Wigan and Leigh locality has a 2.8% BAME demographic compared to 14.1% in England and Wales. However, Wrightington, Wigan and Leigh does have a similar rates of males (49.89% versus 49.43%) and average age (42.1 versus 40.2) to that in England and Wales [26].

**Table 3:** Demographics of referrals

Victim demographics	Year 1 1st May 2018 – 31st March 2019 (***n*** = 319)		Year 2 1st April 2019 – 31st March 2020 (***n*** = 619)^**b**^		COVID-19 1st April 2020 – 31st August 2020 (***n*** = 358)		Other IDVA referrals^**a**^ (***n*** = 3556)	
Gender								
Male	49	13.54%	78	12.60%	47	13.13%	130	3.66%
Female	270	86.46%	541	87.40%	311	86.87%	3381	95.08%
Age^b^								
Under 16	0	0.00%	1	0.18%	0	0.00%	13	0.87%
16–19	24	7.52%	34	6.19%	27	7.54%	202	5.68%
20–39	191	59.87%	293	53.37%	177	49.44%	2415	67.91%
40–59	56	17.55%	140	25.50%	95	26.54%	800	22.50%
60+	48	15.05%	81	14.75%	59	16.48%	108	3.04%
LGBT	–		7	1.13%	5	1.40%	78	2.19%
Learning Disability	–		12	1.94%	1	0.28%	40	1.12%
BAME	–		7	1.13%	3	0.84%	574	16.14%

*Notes*: ^a^Safe Lives (2019)

^b^Age bands provided for 549 referrals in Year 2; age bands for SafeLives IDVA demographics differ slightly (Under 18, 18–20, 21–40, 41–60, 61+)

Not provided in data by WWL NHS Foundation Trust

Referral sources are shown in Table 4. During the first 2 years, the majority of referrals to the service came from A&E (58%). Other areas of the Trust making referrals were: midwifery services, the Walk in Centre, drug and alcohol, mental health and children’s services. 5% were self-referrals. During the COVID-19 period, the referral sources remained broadly in line with the previous 2 years. Referrals from the Walk in Centre increased, from 2 to 6% during COVID-19, whilst those from midwifery reduced (3% during COVID-19 compared to 10% in year 2). The rate of referrals that were repeat referrals was low prior to COVID-19 (3%) and there were no repeat referrals during the COVID-19 period.

**Table 4:** Referral source March 2018 to August 2020

Referral source	April 2020 to August 2020	% of referrals	May 2018 to March 2020	% of referrals
A&E	211	58.94%	544	58.00%
Leigh walk-in	22	6.15%	17	1.81%
Self	15	4.19%	43	4.58%
Midwifery	10	2.79%	90	9.59%
Alcohol Nurses	8	2.23%	26	2.77%
RAAMHT	7	1.96%	36	3.84%
Community Referrals	6	1.68%	26	2.77%
Children’s safeguarding nurse	6	1.68%	6	0.64%
Rainbow Ward	5	1.40%	9	0.96%
Wrightington hospital	4	1.12%	0	0.00%
Repeat	0	0.00%	31	3.30%
Other	64	17.88%	110	11.73%
**Total**	**358**	**100.00%**	**938**	**100.00%**

*RAAMHT* Rapid All Age Mental Health Team, *Rainbow Ward* Children’s inpatient ward

Overall, referrals to the service continued to increase, with a slight drop during April 2020, but a sustained rise thereafter. These findings suggest that demand for the service increased, including during the COVID-19 period and that the IDVAs were able to maintain their response. Referral sources during the Covid period stayed broadly in line with the previous 2 years. During year 1, referrals of males and people aged 40 and over were proportionally higher than at other IDVA services. The proportion of males at the Trust stayed the same over time, whilst the proportion of people aged 40 and over continued to increase. This shows a continued pattern of the IDVAs supporting a different demographic of victims than at other services.

The service reported outcomes for those referred to the service during the period May 2019 to August 2020, these are provided in Table 5. The majority of referrals result in support being provided by the HIDVA (72% pre-COVID-19, 76% during COVID-19). A range of other services are referred to including the multi-agency risk assessment conference (MARAC), refuges, community IDVAs, and Adult and Child Social Care (see Table 2 for more detail about the MARAC).

**Table 5:** Reported outcomes of referrals between 1st April 2020 to 31st August 2020

Outcome	Volume April 2020 to August 2020	Share of referrals (%)	Volume May 2019 to March 2020	Share of referrals (%)
Support	261	75.87%	405	71.68%
Unable to establish contact	37	10.76%	48	8.50%
MARAC referrals	18	5.23%	46	8.14%
Referral to refuge	8	2.33%	9	1.59%
Referral out of area	6	1.74%	5	0.88%
Support from Community IDVA	5	1.45%	2	0.35%
Declined support	5	1.45%	35	6.19%
Adult Social Care referral	3	0.87%	6	1.06%
Child Social Care referral	1	0.29%	2	0.35%
Application for civil orders	0	0.00%	7	1.24%
**Total**	**344**		**565**	

The number of outcomes do not align to the volume of referrals due to the difference in time periods captured for the data and due to some victims still being within the service

### Qualitative findings

Eleven interviews were conducted, with hospital-based IDVAs and other staff with experience of the service. The interviews lasted between 13 and 77 minutes, with a mean length of 46 minutes. Participants’ roles included IDVAs, nursing, health visiting and senior management and they worked in various teams and locations including safeguarding, drug and alcohol services, the walk in centre, unscheduled care and inpatient wards.

Our initial analysis explored the context in which the hospital-based IDVA service was implemented and identified facilitators and barriers to its operation. Interviewees agreed that it was an important resource, addressing previously unmet need, including the identification of types of victim underrepresented in DVA services; men, older people and members of Trust staff. There was consensus that the service was running effectively and meeting its goals. Key enablers identified were: the commissioning and delivery model with the IDVA directly employed and managed within the Trust; engagement and relationship building across the Trust, at both strategic and individual levels; the independent, advisory nature of the IDVA role and interpersonal skills of the IDVAs.

The quantitative analysis showed that demand for the service continued to increase during the COVID-19 period and that the IDVAs were able to meet this demand. Consequently, our analysis of the qualitative data focussed on experiences of providing the service during the pandemic. Three themes were identified: demand for the service during lockdown, the importance of effective working relationships and adapted ways of working.

#### Demand for the service during lockdown

The IDVAs and service manager observed a reduction in referrals to their service, at the start of lockdown, which they attributed to the ‘stay at home’ order and linked to the wider issue of reduced attendances at hospital. Concern was expressed about a likely increase of DVA and victims being trapped at home with perpetrators, unable to access healthcare. However, after this initial drop, activity soon increased again and the service became very busy:

*Yeah, at the beginning it dipped, because people were frightened of coming to the hospital, when it first … went into lockdown...so Marchs’ figures were down, and then April’s just went straight back up again. (IDVA A)*

*People will say ‘it’s been the stress of being under each other’s feet for so long; usually we will see each other for a couple of hours at night, but we’ve not been able to get away from each other’. Normally you can walk away from an argument; during lockdown you couldn’t... That’s what [victims are] saying when we’re speaking to them … And now that lockdown is easing and they can get out this is when they’re all coming through now. (IDVA B)*

The quote below illustrates some of the situations faced by people who were referred to the service at that time:

*because of the pandemic … we're expecting, a mushrooming of domestic abuse and sexual abuse … It's particularly busy [during] the last two weeks … In fact we've had a very wide range of cases come through … sexual abuse and physical abuse, coercion control, people just frightened of going home, don’t want to go back to living that way. Some members of staff have had date rape. They’ve had historical sexual abuse. We've had all of this in the last two weeks. (Safeguarding manager)*

The IDVAs observed an initial drop in referrals at the start of lockdown, followed by an increase and found themselves responding to various challenging situations.

#### The importance of effective working relationships during lockdown

Although levels and intensity of work were challenging, there was consensus that the service had been able to absorb the demand. In fact the continued demand was considered a sign of success, because it showed that victims were still successfully being identified and referred, despite social distancing restrictions. The key enabler here was deemed to be the strong working relationships underpinning the operation of the service – these had been fostered, strategically, by the employment arrangements whereby the IDVAs were based in the safeguarding team, which works across the Trust and efforts of the IDVAs to get to know staff across the Trust, visiting departments and wards in person, described as ‘walking the floor’, which resulted in the IDVAs being a known presence across the Trust. The interviewee quoted below emphasised the value of the IDVAs being known in person:

*… the face to face aspect has been massively successful … the minute that we pull away from that face to face is the minute that you will see staff will disconnect from that and they won't make the referrals the same … like out of sight out of mind … As a healthcare system we still actively promote, you know; ‘you must look out for safeguarding … you must refer’. But it really does help if you have that face … You're more likely to ring for advice if you know who you're talking to and you’ve got that relationship rather than just ringing an anonymous person or going online. (Matron, unscheduled care)*

The perception of the IDVAs as a strong presence, across the Trust, which endured, even through lockdown, when face to face contact was reduced, was reinforced by the IDVA quoted below. Here, the IDVA, recounts a discussion with a counterpart (another IDVA) working at a neighbouring Trust, who reported that levels of referrals had reduced to and stayed at a very low level:

*… some of the other [areas] that have got IDVAs now, in hospitals and they’re not NHS employed they’re seconded in … Their caseloads, they’re not getting as many through [since lockdown]. I had an email from [a hospital-based IDVA at a different hospital], and she’s like ‘through Covid, I’m actually helping another department, ‘cause there’s no cases coming through, how are you finding it?’ And I’m like, ‘we are absolutely snowed under’. So, what are they doing different to us? They’re not getting out there. We’re out there … we’re going to A&E every morning, we go down to the wards. We’re out and about all over the hospital. And we’re being seen, so staff recognise us. When I walk onto a ward, staff say, [name]‘s here, that domestic abuse woman’s here. You know?...So, you’re at the forefront of them every day, so when someone comes in and discloses, they know, oh we’ll phone Safeguarding and get [an IDVA] down. And that is the key. (IDVA A)*

The sustained demand for the service was seen as a function of the presence of the IDVAs, even during lockdown, when in person contact could not be maintained; this was attributed both the structure of the employment arrangements and to the efforts of the IDVAs to build awareness of the service across the trust and develop strong working relationships.

#### Adapted ways of working during lockdown

The social distancing required during lockdown brought several changes to the referral process. The IDVAs adopted various workarounds, such as conducting the risk assessments over the phone from a different room and using PPE when visiting wards. There were also changes to the ways that abuse was identified and disclosed. Before lockdown, inpatients were referred to the IDVAs after staff witnessed abuse by family members during visits. When visitors were not allowed, naturally this did not happen, but instead, some inpatients made disclosures to staff on the wards, which was considered only to have happened due to those patients being alone, without visitors.

Disclosures from staff at the Trust, who were experiencing DVA themselves, began happening soon after the service was established. During lockdown, staff had have regular COVID-19 tests at the Trust testing centre and could only attend alone; the centre became a new location where disclosures happened:

*So, it was our staff that was disclosing … to the … nurses that are testing them to see if they’ve got Covid … ‘Cause you can’t go to the Covid test with somebody else, you have to go on your own [so they could] speak to somebody and say, ‘this is my only chance to get out, and this is what’s happening’. (Safeguarding manager)*

Speaking to (suspected) DVA victims alone was a familiar challenge to the hospital-based IDVAs, as some perpetrators try and stay with the victim constantly, including during healthcare consultations. The IDVAs noted that social distancing restrictions made it easier for them to see some victims alone:

*Normally we would aim to try and get them into the clinic … If they’re due to come for an appointment … we’ll sit in a separate room just in case the partner comes. And the health visitor will then say [to the partner], ‘oh we just need to take you in there but it’s only a little room, so you wait in the waiting room’. I mean, COVID has been good in one way because they’ve not been allowed to actually come with them or go into the rooms with them so it’s given them that space really, that opportunity without the partner being there. (IDVA B)*

Finally, before COVID-19, the MARAC was held weekly, at the local police station. (See Table 2 for detail about the MARAC) In lockdown, the MARAC was switched to daily, remote (telephone) meetings. The IDVAs found the more frequent contact with the MARAC beneficial, both for their working relationships with the other members and and also, they felt potentially for the safety of victims, by enabling a quicker response. The IDVAs also found the virtual meetings more convenient and time-efficient and hoped these would continue permanently.

Lockdown affected the working of the service in various ways. Social distancing requirements necessitated various workarounds and brought some changes to the ways that abuse was identified and disclosed. Some unexpected changes were deemed beneficial; sometimes it was easier for IDVAs to speak with victims alone and the MARAC meetings became more efficient and potentially part of a quicker, safer response to victims.

## Discussion

### Summary of main findings

We conducted a mixed methods evaluation of a hospital-based IDVA service. The evaluation coincided with the early stages of the COVID-19 pandemic, a situation where DVA became an increasing concern. This coincidence prompted us to explore and understand the impact of the pandemic on the IDVA service and how the service responded. This paper assesses the activity in the hospital-based IDVA service during the COVID-19 pandemic and addresses the research questions: What was the demand for the service? How did the service respond? What facilitated this response? We used quantitative methods to explore referral activity before and during the pandemic and qualitative methods to understand the processes involved in providing the service. The quantitative analysis showed that referrals dropped slightly at the start of lockdown, then increased and continued to rise; the qualitative findings reiterated this pattern. Referrals came from departments across the Trust, the majority from A&E. Pre-pandemic, the population supported by the service included higher proportions of males and people aged 40 and over than at other IDVA services; this continued to be the case during the pandemic. The qualitative findings indicated an efficient, flexible response during the pandemic, enabled by strong working relationships and by using workarounds.

### Discussion and comparison with existing literature

The quantitative and qualitative analyses showed that demand for the service dropped at the start of lockdown. Interviewees interpreted this drop as due to lockdown and fewer victims attending the Trust, rather than a reduction in occurences of DVA. Indeed, concern was expressed that lockdown was particularly problematic for DVA victims confined in close quarters with their perpetrators and therefore at greater risk, whilst also being less likely to leave home and seek help. These findings echo others’ concerns about DVA victims during lockdown [18], and evidence of decreased hospital admissions for DVA, despite social conditions that increase the risk of violence [27].

After the initial drop in referrals at the start of lockdown, both the quantitative and qualitative findings showed a busy service with increased referrals; the IDVAs continued to provide an efficient service, taking on and supporting all cases referred to them. This is notable, particularly considering that DVA is often a hidden problem and detection relies on staff retaining focus on the signs and making sensitive enquiries; staff at the Trust continued to this during lockdown, with intense pressure on hospital services and severe disruption to usual methods of communication. The use of the service depends on multiple factors, including demand and available and accessible supply. Given that provision had remained equivalent to pre-pandemic rates (2 FTE IDVAs) and reduced attendances in hospital ED and inpatient activity [28] the increase in referrals appears to reflect an increase in need for the service and the number of victims presenting at the Trust. There was consensus amongst our interviewees that the IDVAs had a strong presence and were known to many staff in person, factors which have been acknowledged as important at equivalent services [12, 15]. At the Trust, considerable effort had been made to foster effective working relationships; strategically, by employing the IDVAs directly, locating the IDVAs within Safeguarding and through the IDVAs’ efforts in getting to know individual members of staff. Building an effective detection and referral system, underpinned by strong working relationships, may have contributed to the sustained referral activity levels. Also, a contrast was drawn with a neighbouring hospital-based IDVA service where qualitative data suggested that referrals had dropped at the start of lockdown, but not risen again. The inference here was that at the Trust, working relationships were such that even during lockdown and social distancing, when IDVAs could not be physically present and visible across the hospital, they were still a strong presence in that staff still remembered and were able to make referrals to them. Furthermore, referrals came from a wider range of departments than at other hospitals Trusts. Taken together, these findings show a service embedded firmly across a large organisation (a NHS Foundation Trust spanning several physical locations) with working relationships strong enough to endure and facilitate an effective service during lockdown.

The quantitative data, showed that the IDVAs supported a different demographic compared to other services [24]; referrals included higher proportions of men and more people aged 40 and over. This had been the case before the Covid-19 pandemic and continued throughout our data analysis period. Several factors could potentially contribute here, for example, staff at the Trust having good skills in recognising signs of DVA and undertaking routine sensitive enquiry with men as well as with women. In terms of the older demographic, awareness of signs of abuse that tend to become apparent during in-patient stays, might have contributed to detection of cases, considering that older people are more likely to have stays in hospital. The findings also indicate a service that broadens access, by supporting populations whose needs may not be identified at other services.

All sectors of healthcare were affected by the COVID-19 lockdown and restrictions; changes to working practices such as using PPE, switching from in-person to remote/telephone appointments – with the patient risk assessments and MARAC - was widespread. In this case, the increased efficiency of remote MARACs was an unexpected advantage. Most patients have been required to attend appointments alone and whilst sometimes unfavourable, this actually benefitted the IDVAs when they wanted to speak with a (suspected) victim alone. The disclosures at testing sites were a further unexpected consequence of social distancing measures.

### Strengths and weaknesses and further research

This is the first study to investigate the provison of a hospital-based DVA service during a pandemic and associated lockdown. Our mixed methods approach allowed us to combine data on referrals and processes. An advantage of researching a population accessing health care in hospital that it allowed for study of people who are unlikely to take part in other research, such as clinical trials or surveys – and in particular allows for inclusion of a higher proportion of those experiencing the most severe harm from DVA [29]. The empirical data relate to one hospital Trust and therefore may not be generalizable to other settings. The quantitative data show activity at the hospital but the equivalent data for other, community IDVA services are not available so it is not possible to gauge the impact on these services. Our wider evaluation included analyses of health service use and costs; these require long-term follow up. A study using a comparative design would allow for a robust quantitative design to determine the size of any causal effect. The qualitative component of the study included the perspectives of staff working in or connected to, the IDVA service, but the experiences of victims of DV referred to the service were not included; a future study could include these service user perspectives.

## Conclusions

The provision of specialist DVA support within hospitals is imperative, as victims presenting at hospital are often those at highest risk. Previous evaluations have shown that hospital-based services can be effective, but also noted shortcomings in terms of locating IDVAs in one clinical area with limited reach across other departments and tensions related to staff not being directly employed in the Trust. The WWL hospital-based IDVA service provided effective support to DVA victims and improved on previous schemes by employing IDVAs within the safeguarding team and through the IDVAs’ efforts to build relationships and a presence throughout the Trust. The scheme appeared robust during the COVID-19 pandemic, during which circumstances (via lockdown) are hypothesised to have exacerbated the need for support for victims of DVA at most risk of severe harms, which hospital-based IDVAs are ideally placed to provide. Other local health economies should note the capacity for hospital-based IDVAs to provide effective and appropriate support for high-risk victims of DVA, and in cases where a similar model is adopted or piloted, can look to the potential improvements the WWL scheme made on previous services. The findings are relevant to policy on access to healthcare They indicate a service that can broaden access by supporting vulnerable, at risk populations whose needs may not be identified at other services. The findings extend the evidence on support for victims of DVA; research on a hospital population allowed us access to a sample that included people who are unlikely to take part in other research, such as clinical trials or surveys – and in particular allowed for inclusion of a higher proportion of those experiencing the most severe harm from DVA [29].

## Data Availability

Data on referrals is sensitive data that is unavailable for access. Insights data is available via the SafeLives website: https://safelives.org.uk/practice-support/resources-domestic-abuse-and-idva-service-managers/insights All authors have completed the ICMJE uniform disclosure form at www.icmje.org/coi_disclosure.pdf and have the following competing interests: funding support from NIHR and from NHS England and NHS Improvement and Wrightington, Wigan and leigh NHS Foundation Trust for this work; WW reports grant funding for other work from the Department of Health Policy Research Programme and NIHR. No other relationships or activities that could appear to have influenced the submitted work are declared.

## Abbreviations

A&E: Accident and emergency department
BAME: Black and minority ethnic
DVA: Domestic violence and abuse
IDVA: Independent domestic violence advisor
MARAC: Multi agency risk assessment conference
